# Physicians’ and nurses’ perspective on chronic disease care practices in Primary Health Care in Brazil: a qualitative study

**DOI:** 10.1186/s12913-022-08078-z

**Published:** 2022-05-19

**Authors:** Carolinny Nunes Oliveira, Marcio Galvão Oliveira, Welma Wildes Amorim, Clavdia Nicolaevna Kochergin, Sóstenes Mistro, Danielle Souto de Medeiros, Kelle Oliveira Silva, Vanessa Moraes Bezerra, Vivian Carla Honorato dos Santos de Carvalho, José Patrício Bispo Júnior, José Andrade Louzado, Matheus Lopes Cortes, Daniela Arruda Soares

**Affiliations:** 1 Master’s Program in Collective Health, Multidisciplinary Health Institute, Federal University of Bahia, Vitória da Conquista, Bahia Brazil; 2 Epidemiology and Collective Health Nucleus, Multidisciplinary Health Institute, Federal University of Bahia, Hormindo Barros Street, 58 - Candeias, Vitória da Conquista, Bahia 45029-094 Brazil; 3 Departament of Natural Science, State University of Southwest of Bahia, Vitória da Conquista, Bahia Brazil

**Keywords:** Primary healthcare, Family health strategy, Hypertension, Diabetes mellitus, Chronic conditions

## Abstract

**Background:**

Primary health care-oriented systems provide better healthcare, especially for chronic diseases. This study analyzed the perspectives of physicians and nurses performing care for patients with chronic diseases in Primary Health Care in a Brazilian city.

**Methods:**

A qualitative study was conducted in Vitória da Conquista, Bahia, Brazil, using semi-structured interviews with five physicians and 18 nurses. The interview included questions from an analytical matrix based on three dimensions of healthcare practices: organizational, technical care, and biopsychosocial, following a deductive approach. The interviews were fully transcribed and analyzed using a thematic categorical approach.

**Results:**

The results indicated that the provision of chronic care occurs in a comprehensive way. Potentialities were identified in the diversification of access, offer of care actions and technologies, integration of teamwork, and bringing together social networks to foster autonomy and self-care. Weaknesses were mostly related to the high number of people in the teams, follow-up of several cases, high turnover of support teams, low integration of Primary Health Care with other levels, difficulties in intersectoral articulation and family participation in care.

**Conclusion:**

The multidimensional assessment of health care practices aimed at individuals with chronic noncommunicable diseases was useful to portray the strengths and weaknesses of the services. It also ratifies the need to consider the importance of and investment in primary health care by offering the necessary technical, political, logistical and financial support to the units, to ensure the sustainability of the actions by nurses, doctors and entire team.

## Background

High morbidity and mortality due to chronic diseases is a global challenge, especially in developing countries [1] where socioeconomic inequalities have a striking impact on risk factors and decreasing access to health services [2]. In 2021, chronic diseases accounted for 73,6% of all deaths worldwide, with cardiovascular diseases and diabetes in the top ten list of most important causes [1].

In Brazil, the burden of chronic diseases remains high despite the implementation of public policies aimed at controlling risk factors, preventing, and promoting health; implemented fiscal austerity measures reduce the role of the state in providing public services focusing on chronic noncommunicable diseases (NCD) [3]. The epidemiological and demographic transitions culminated in changes in the patterns of illness and increased life expectancy, implying an increased load of NCD, that will need to be coordinated by health professionals to provide higher-quality chronic disease management [4].

Chronic diseases pose a considerable challenge for local health systems. Primary health care (PHC) demands the development of care practices aimed at the early identification of people with risk factors and disease, risk stratification, promotion/prevention/assistance interventions, regular follow-up of health conditions, treatment adherence encouragement [5–7], in addition to health care coordination and patient screening in the health care network (HCN) [8]. The PHC oriented health systems provide chronic disease care in terms of resolution, equity, and cost–benefit, as they improve access, and avoid redundant exams, consultations, procedures and hospitalizations [2, 9].

In Brazil, inspired by the Alma Ata conference for the organization of primary care, healthcare practices have been highlighted with the advent of the Unified Health System (*Sistema Único de Saúde*-SUS) in the 80 s and the Family Health Strategy (FHS) in the 90 s, increasing the role of PHC in restructuring, strengthening, and rationalizing health systems [9]. Furthermore, free access, territorial focus, orientation to individual/family/community health needs, providing a range of accessible and resolutive services to regularly and continuously meet the population’s needs, and efficient healthcare coordination between PHC and other levels of care are some PHC governing principles in the country [10].

Although PHC services may be better positioned to address the prevention and management of NCD, other deficiencies in primary healthcare practices have been identified in many studies [10–13]. In Brazil, despite efforts to address disease burden, this issue is perpetuated by insufficient funding, human and material supply chain issues [13], and fragmented health systems [7, 14–16]. In this way, the approach to chronic diseases is multifaceted as it involves several dimensions (organizational, technical care, psychosocial), which are often difficult to consider together in health research [13]. Qualitative methodologies have been widely used to obtain a better contextual understanding of health services and how they impact on chronic conditions [17], as well as to address perspectives related to the practices of health professionals in PHC services, giving an indication gaps in care delivery. This study analyzed the perspectives of physicians and nurses performing care for patients with chronic diseases in Primary Health Care in a Brazilian city.

## Methods

### Study design

This is a qualitative study that maps a multidimensional scope of chronic disease care practices in the context of the FHS in Brazil.

### Study setting

**Fig. 1:**
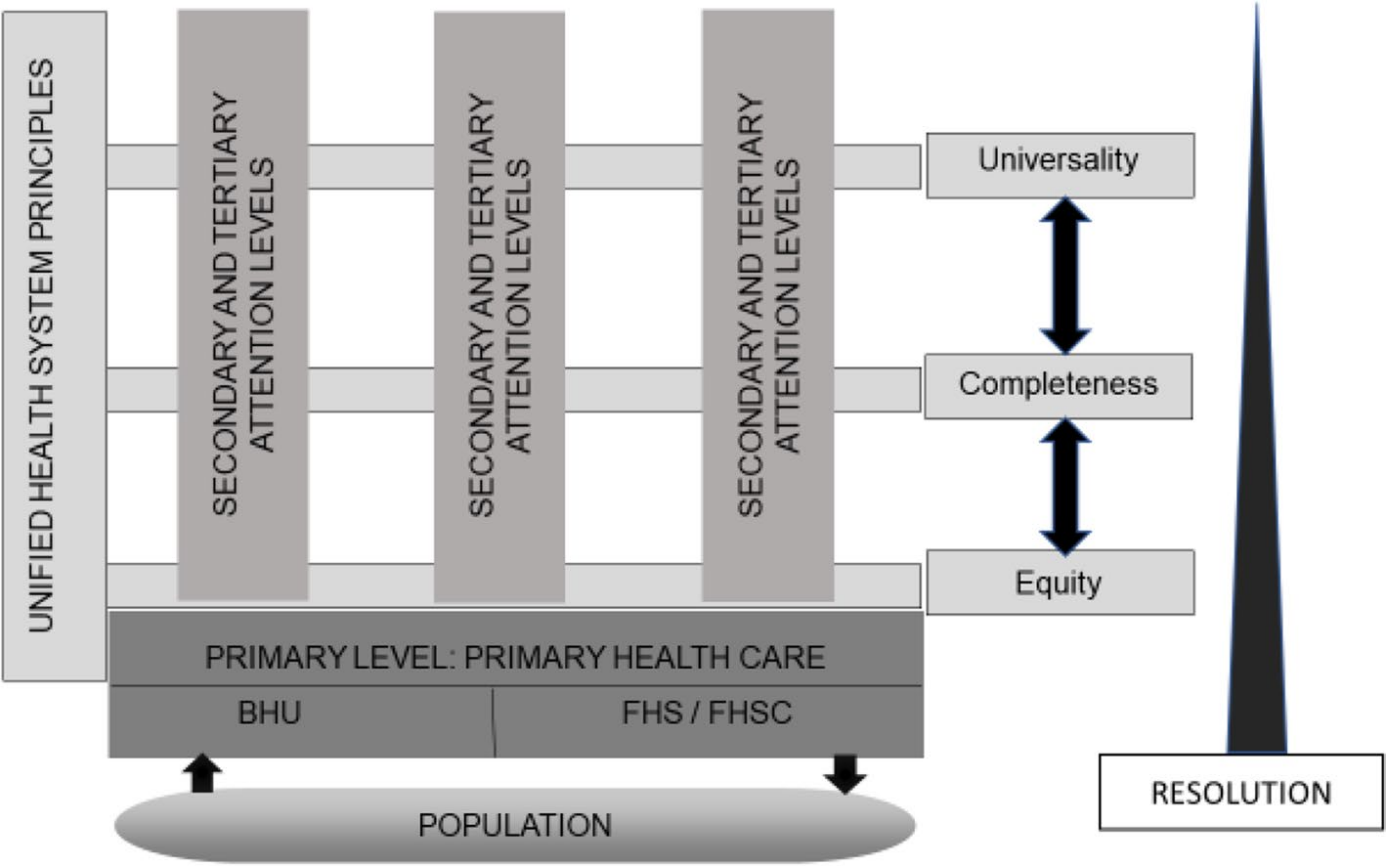
Hierarchical organization of the Unified Health System in Brazil and the relationships between levels of care. The figure illustrates the hierarchical organization of the Unified Health System (SUS) and the relationships between levels of care. On the right side are the SUS principles that cover all levels of health care, bidirectional arrows show this interdependence. At the bottom, it is shown that the population’s demands must reach the primary level, contained in the shaded rectangle and are directed to health units with the Family Health Strategy (FHS) or to the Basic Health Unit (BHU). The main difference between the units is that the first has a defined territory and population, and operates under the logic of teamwork, while the second does not. Support actions by the Family Health Support Center (FHSC) contribute to increasing the scope of FHS actions and improving health conditions. It is indicated that PHC can solve about 80% of the population’s demands by coordinating the care of people between the levels of care, as shown in the triangle on the right

The study was conducted in Vitória da Conquista, BA, Brazil, an important economic and educational nucleus. This city is the medium-sized in relation to Brazil and head office of the Health Region of Southwest Bahia, which includes 19 other cities, and serves as a focal point for secondary and tertiary health service delivery. It has a medium socioeconomic development, but still marked by many disparities in terms of income and schooling. The estimated population is 306,866, a human development index of 0.678, gross domestic product per capita of BRL 20,761.05, and 238 health establishments, of which 48 are health units; of the 48 health units, 35 health unit are in the FHS model [18]. The organization of the Unified Health System in Brazil can be seen in Fig. 1.

These health units have 29 FHS in urban areas and 19 in rural areas, comprising approximately 48% of the population, and representing the main Brazilian model of primary health care. These health units emphasize health care in community health facilities and at home to a defined local population, having about 2,000–3,500 people registered. The health teams include physicians, nurses, dentists, nursing technicians, and community health workers (CHW). In addition, they have the assistance of multidisciplinary family health support centers (FHSC), composed of specialists such as physiotherapists, nutritionists, psychologists, and physical educators. Each FHSC is responsible for supporting five to nine FHT teams in an integrated and collaborative way with activities of a technical-pedagogical or clinical-assistance nature and, therefore, has a specific coordinator not linked to the health units.

### Participant recruitment

A total of 10 FHS in urban areas were intentionally chosen owing to their easy access for presenting specific work processes when compared to rural units, and because this study was nested in a larger study entitled “Vitória da Conquista Health Rise Project” whose central objective was to evaluate the effects of health interventions on high blood pressure (HBP) and diabetes mellitus (DM) control in patients treated at the SUS. Thus, when using the term chronic diseases in this study, we are referring to HBP and DM.

Purposive sampling was used to recruit physicians and nurses. These professionals were chosen owing to the comprehensive and diversified scope of their practice in chronic diseases in PHC, which is consistent with the objective of this investigation. As a criterion in the study, professionals with at least one year of experience in a unit in an urban area were included. Professionals on leave at the time of the interviews were excluded.

### Data collection

The data were collected in four months: October to December 2018 and February 2019. Professionals eligible to participate in the research were contacted by telephone, using a list obtained from the health department. The interview was scheduled at a time and place chosen by the participant. Before the interview, the objectives of the study were explained, and the participants signed an informed consent form. The interviews were audio recorded and subsequently transcribed verbatim.

Most interviews were conducted at the health units, during lower patient flow (beginning or end of the working day), in a private and quiet space. A total of 23 semi-structured interviews were conducted with physicians (PHY) and nurses (NUR) by a researcher trained in qualitative research methods. This study was approved by the ethics committee. A pilot study was previously carried out, it sought to test, evaluate, review and improve research instruments and procedures. Four professionals were included to participate. With the application of logistical procedures, the estimated duration of the interviews and the best time to approach the professionals (in this case, beginning of work) were identified. Regarding the data collection instrument, there were no substantial changes, other than making the language clearer in order to facilitate understanding. The analysis followed the same perspective described in the article and facilitated the training of the researcher in the establishment of categories and organization of data, confirming the feasibility of the research.

The number of interviews was defined using the sample-saturation criterion when, in the researcher’s assessment, the data obtained contained sufficient elements to interrupt the collection [19]. The most important criterion for interrupting the collection was based on the researchers’ continuous judgment regarding the availability of data that sufficiently showed variety and depth to understand and represent the thematic categories and respond to the outlined objectives. Repetition of words and/or terms in the coding and categorization process was also considered. The interviews lasted from 21–58 min.

### Data analysis

A matrix was developed and constituted a dynamic and interactive process of research in national and international literature, integrating the author’s experience. There were three steps in the analysis framework. First, methodological sources were chosen that address the characteristics of comprehensive PHC, as well as the guidelines and strategies for the care of people with chronic diseases [14, 20–25]. Second, the main dimensions related to care practices aimed at people with chronic diseases in PHC were identified. Organizational, technical care, and biopsychosocial aspects were considered. Third, an interactive process of systematization and refinement of the dimensions of the study and its corresponding components was carried out, based on the differences, similarities, and intersections.

Regarding the matrix, the organization dimension relates to how the work process is structured by the health care teams involved [14, 15, 20]. The technical care dimension includes the skills professionals require to respond to the users’ problems/needs and their ability to connect to them [21, 22]. The biopsychosocial dimension includes psychosocial elements and community action [20, 23, 24] to develop health awareness among popular organizations. The matrix can be found in Table 1.

**Table 1:** Analysis matrix of healthcare practices for chronic diseases in the Family Health Strategy

Analysis dimensions	Components
Health care practice organization	- Schedule and access organization
	- Interdisciplinary action with FHT and FHSC
	- Articulation between care levels
	- Action planning and monitoring according to health indicators
Technical care aspects of health care practices	- Broad provision of promotion, prevention, and health care services aimed at people with chronic diseases
	- Use of care protocols
	- Information exchange and case discussion in the multidisciplinary team
	- Establishment of a bond in care practice
	- Presence and use of plans/strategies for periodic follow-up and management
	- Consistency in entering data in the EMR
Biopsychosocial approach to care practices	- Knowledge of the socio-epidemiological profile of the coverage area
	- Intervention actions on social risks and vulnerabilities in the territory
	- Promotion of autonomy and self-care development in people with chronic diseases
	- Presence and role of the social support network

*FHT* Family Health Team, *FHSC* Family Health Support Centers, *EMR* Electronic Medical Record

Thematic content analysis followed a deductive approach and included the following stages: superficial and exhaustive reading of transcripts to search for repeated patterns and intra- and inter-group contradictions carried out independently and jointly among the researchers (these contradictions were resolved by consensus to have the first classifications); material exploitation from coding, classification, and categorization procedures; and results interpretation based on the analytical matrix [25].

## Results

The participants were predominantly women (*n* = 21) aged between 31 and 45 years (*n* = 16) and brown (*n* = 16). Most (*n* = 10) had worked for up to five years in FHS, and had a specialization in collective health (*n* = 15). These characteristics generally followed the same pattern among the professional categories represented, and can be seen in Table 2.

**Table 2:** Distribution of sampling characteristics of Physicians and Nurses, Vitória da Conquista, 2019

Variables	Health professional		Total (n)
	Physician (n)	Nurse (n)	
Age			
18 to 30 years	01	01	02
31 to 45 years	03	13	16
> 46 years	01	04	05
Gender			
Women	04	17	21
Men	01	01	02
Race			
White	01	11	12
Black/Brown	04	12	16
Time of work in FHS			
< 5 years	01	09	10
6 to 10 years	03	06	09
11 to15 years	01	08	09
Specialization in collective health			
Yes	03	12	15
No	02	06	08

*FHS* Family Health Strategy

The results were organized in theoretical categories corresponding to the three dimensions and the correlated components contained in the analytical matrix. The most illustrative reports in each category are shown in Tables 3, 4, and 5, and the weaknesses and potentialities related to the practices are summarized in Table 6.

**Table 3:** Organization of chronic disease care practices

Components	Illustrative discourse excerpts
Schedule and access organization	(NUR 1) “(…)the screening happens at the entrance door, if the patient cannot be seen that day (…) there is a schedule, then that patient, when he comes, gets a response either that day or another day, according to his demand.” (NUR 7) “(…) there is a monthly quota (for specialists) (…) it does not supply the demand, so there is no good access.”
Interdisciplinary action with FHT and FHBCSC	(NUR 2) “(…) I discuss (the cases) with the doctor and there are groups where we can define actions. In the same week (within the group) we have team meetings, and eventually we discuss cases from that group, at the team meeting and we can discuss clinical cases as well.” (NUR 4) “(…) they (FHSC professionals) are always like this, only a representative at the meeting, we end up discussing (clinical cases). I cannot say that I have already witnessed construction of a therapeutic process, it may exist, but I did not participate (…).” (NUR 16) “(…) the issue of going to a consultation and not having the prescription renewed, of not having a specific exam request, a referral to a specialty, is still very much present.”
Articulation between assistance levels	(NUR 6) “(…) I think referral and counter-referral for hypertension and diabetes, does not exist in reality. Unfortunately, the only referral that we make is related to the reason for referral that you describe well. There is also no counter-referral feedback, if he (patient) does not come back to us and says what happened, we will have no knowledge (NUR 16) “(…) it would be good if the EMR was an integrated system (with other levels of complexity).”
Action planning and follow-up according to health indicators	(PHY 4) “(…) our (team) meetings are weekly, we discuss these cases (of hypertensive and diabetic patients) and always have extraordinary meetings at the beginning of the shift with (health) agents mainly (NUR 1) “(…) (follow-up) by the team does not (occur), I would say that this action is more individual, they (physicians) are concerned about actually seeing if the patient has returned, they immediately signal for health agents to keep an eye on it.” (NUR 4) “for action planning we mainly use information from the CHWs, right, we are based on this mostly, the need, the demand comes mostly from the CHWs.”

*FHSC* Family Health Support Centers, *CHW* Community Health Workers

**Table 4:** Technical care aspects of chronic disease care practices

Components	Illustrative discourse excerpts
Providing a broad portfolio of promotion, prevention, and healthcare services aimed at people with HBP and DM	(NUR 15) “(…) there is blood pressure verification (…) capillary glycemia, we have the glycated hemoglobin device (…) immunization (…) health education groups (…) medical consultation, nursing consultation, consultation with the FHSC team (…),we also have the support of community health workers, home visits (NUR 3) “(…) for hypertensive patients, we offer two groups (educational) (…), in addition to consultation with the medical professionals and myself (nurse) and care (…), which is when I see the patients referred by her (physician) or (…) I guide the HBPM device. Moreover, we visit (homes) (…). Regarding diabetics, we have a group (educational) in the month that is for insulin-dependent and diabetic patients (…).”
Use of care protocols	(PHY 10) “(…) (I use clinical protocols) of course, (…) the question of when the medication will begin immediately, when it is going to be a diet only, in the case of diabetic patients, when to start insulin or hypoglycemic medication, we use all of this.”
Information exchange and case discussion in the multidisciplinary team	(NUR 11) “(…) there is a direct contact with the community health agent and we often do this, the discussion of cases in team meetings always take place when necessary.” (PHY 3) “(…) Nurse technicians always point out patients who probably have hypertension or diabetes and who have not yet been diagnosed so that we can keep an eye on them. The health workers point out, direct, speak about patients who are not presenting adherence, so there is good rapport in the team.”
Bond establishment and welcoming in care practice	(PHY 5) “(…) so, I won’t tell you we can listen to the patient calmly, because we can’t, the demand is high, so I try to get most problems the patient is bringing to me, but more time with the patient would be better.” (NUR 5) “(…) we began visiting (home) these patients (hypertensive and diabetic) and on the visit I schedule the consultation and establish a bond.”
Presence and use of plans/strategies for periodic follow-up and management	(PHY 2) “(…) I try periodic follow-up and case management with severe patients, decompensated patients. I don’t follow-up with others who are compensated.” (NUR 13) “(…) with all of them (patients) the return is on demand, when they think they need to come they come, the prescription is always renewed, because the obstacle is not distributing the medication.”
Consistent data entering in the EMR	(PHY 4) “(…) the EMR is awful. It is a long chart, redundant, repetitive (…). We have Internet instability in the city and frequent EMR updates from the health ministry. It also has a bad interface, it is not presented first when you are seeing a patient, it already opens in the clinical part, then you do not see previous comorbidities, previous medications, previous clinical history, so its organization is awful (NUR 11) A difficulty (with EMR) that is unbearable is to enter all exam data, I think a lot of record is lost (for this)

*HBP* High Blood Pressure, *HBPM* Home Blood Pressure Monitoring, *DM* Diabetes Mellitus, *FHSC* Family Health and Basic Care Support Centers, *EMR* Electronic Medical Record

**Table 5:** Psychosocial approach to chronic disease care practices

Components	Illustrative discourse excerpts
Knowledge of the socio-epidemiological profile of the area	(PHY 10) “(…) is a population with a low economical level. Sometimes they have a difficult educational understanding, they do not understand the issue of medication. (…) our territory is a peripheral area so all this definitely influences care.” (NUR 1) “these patients have low educational level, are older, patients that we notice. They live in broken families or alone, patients who have associated psychological problems basically make healthcare very difficult.”
Intervention actions on social risks and vulnerabilities in the territory	(NUR 13) “If we had time, we could do a much better job, there is this woman who participates in all our activities, and her blood pressure is never controlled, (…) then we began to follow her up individually. Then I discovered that it is because her son is involved in drug trafficking. Every time there is a police raid, her pressure goes up and no medication can control it. She needs another device to help her, now, imagine doing this with six hundred (hypertensive patients). Today, after follow-up, her pressure is 120/80.” (NUR 3) “(…) most of them (hypertensive and diabetic patients) are older, many live alone or live with a partner who is also older and one helps to care for the,(…), so I have to give more attention to these patients (…), follow them up closely.”
Promotion of autonomy and self-care development in HBP and DM patients	(NUR 2) “We try to make him (patient) understand well, at least the process of non-medicated and medicated treatment, we try to talk, show, we even draw if needed, (…) so we try in every way to stimulate self-care.” (PHY 10) “I always consider the issue of care, changes in habits, always the issue of food, weight loss, taking the right drug, doing physical activity, self-care, stress, so, they can change things, right?”
Presence and role of social support network	(NUR 3) “(…) here in the neighborhood there is the community room that is always available, and in this sense the community here also helps a lot, they are always ready to organize (…), make things happen.” (NUR 20) “(…) you don’t have much family participation because most family members work, and elderly patients spend the whole day alone. So, it is difficult, and when the family is present at the time of care or visit, they even listen to the guidelines, but they are not active in participating in this care process.”

**Table 6:** Potentialities and weaknesses of chronic disease care practice

**Dimension: Care practice organization**		
**Components**	**Potentialities**	**Weaknesses**
Schedule and access organization	- Diversification access strategies	- High number of people with chronic diseases per FHT for follow-up - Monthly quota of specialists insufficient to supply demand of people with NCD
Interdisciplinary action with FHT and FHSC	- Collaborative practices among professionals in FHT	- Big FHSC turnover with the FHT
Articulation between assistance levels	- Computerization and integration of communication in the FHS	- Restricted integration with other levels of care
Action planning and follow-up according to health indicators	- Filter for requesting exams and procedures optimizing resources	- Follow-up not based on indicators
**Dimension: Technical care aspects of health care practices**		
**Components**	**Potentialities**	**Weaknesses**
Offering a broad portfolio of health promotion, prevention, and care services	- Provision of several promotional, preventive, and care services to people with chronic diseases - Provision of new care technologies	-Division of labor according to professional category
Use of care protocols	- Knowledge of care protocols by all professionals	- Professional segmentation in the use of care protocols
Information exchange and case discussion in the multidisciplinary team	- Team meetings held constantly - Good rapport and sense of responsibility between professionals	- Difficulty in contacting and discussing cases with FHSC professionals
Bond establishment and reception in care practice	- Recognition of the importance of bonding and receive to improve care practices	- High number of people makes it difficult to bond and receive chronic disease patients
Presence and use of plans/strategies for periodic follow-up and management	- Use of diversified case management mechanisms	- Reactive follow-up with a focus on severe case management
Consistent data entering in the EMR	- Large-scale use of the EMR - Possibility of using care indicators to promote improvements in the provision of care	- Poor Internet network quality - Poor EMR configuration and interface for viewing and inserting data - Non-integration of the EMR into the care network
**Dimension: Biopsychosocial approach to care practices**		
**Components**	**Potentialities**	**Weaknesses**
Knowledge of the socio-epidemiological profile in the registered area	- Recognition of user vulnerability profile for NCD	- Difficulty in action due to the socioeconomic problems of people with chronic diseases
Intervention actions on social risks and vulnerabilities in the territory	- Broader professional vision to overcome risks and vulnerabilities	- Difficult intersectoral articulation
Promotion of autonomy and self-care development	Recognition of the importance and availability of health education activities in different modalities in all FHTs	Not identified
Presence and role of social support network	- Recognition and establishment of partnerships with actors and social support devices in the territory	Difficult articulation with the family for assistance in providing care

*FHT* Family Health Team, *FHSC* Family Health and Basic Care Support Centers, *EMR* Electronic Medical Record

### Health care practice organization

For the interviewees, the organization of care practices towards people with NCD occurred in a diversified way, considering mechanisms that are institutionalized in the municipality, but also based on specific arrangements made by the investigated units.

Access was predominantly based on spontaneous demand at the unit’s reception (*acolhimento*) or granted by the screening service. From the reception, professionals listen to problems, assess symptoms and understand the case, even if the problem presented by the user is not resolved immediately. With triage, it is possible to select who will be seen at the PHC on the same day (as in urgent cases) or referred to specialists based on quotas.

For referral to specialists, all health units follow the same pattern: they receive quotas from a Consultation Scheduling Center-CMC, they select patients for vacancies and the scheduling is decentralized. However, despite the autonomy of health units to schedule appointments with specialists, there are too few specialists to service the local population requirements, especially those related to cardiological, endocrinological and ophthalmological evaluations (Table 6).

Other access strategies for consultation and examination access are organized through health education groups, at which time some teams collectively select eligible individuals to carry out short-term consultations and exams, in addition to renewing prescriptions (Table 3, NUR 1).

The high prevalence of chronic disease cases in the territories was also indicated as a contributing factor hindering access, given the difficulty of the team in following up (Table 6).

The organizational arrangement between FHT and the organization of interdisciplinary work with the FHSC team is expressed in horizontal and partnership relationships in the planning of actions and health care. However, the greatest responsibility for planning falls on the FHT due to the high turnover between the supported units. (Table 3, NUR 4). Nevertheless, FHT interaction was well organized (Table 3, NUR 2), despite reports that indicate a greater valuation of including individual care performed by the physician, and the high number of procedures and medications requested by the users (Table 3, NUR 16) taking time away from their participation in organizational responsibilities.

Regarding the articulation of the FHS with the care network, the referral and counter-referral communication mechanism was reported as a one-way process in which the referral occurs only from the FHS to other levels, in a non-standardized way (Table 3, NUR 6). Despite the electronic medical record (EMR) being fully inserted in the FHT routine with shared access to the information recorded, it has not been able to reduce networking issues as it is not yet integrated with other health care levels (Table 3, NUR 16), constituting a barrier to the continuity of care.

The planning of activities developed by the service is based on the demands and information collected from the FHT team, especially from the CHW (Table 3, NUR 4). Monitoring was also reported to occur in a more punctual and individual form, mainly by the physician (Table 3, NUR 1), which proved to be important for the optimization of requests for exams and procedures, and the prioritization of care.

### Technical care aspects of health care practices

The interviewees reported a broad range of services and activities provided for chronic disease patients, including health educational group and health-promoting actions for users, prevention of complications in previously diagnosed cases, screening and diagnosis of new patients, healthcare through consultation requests and scheduling, as well as examinations at the same level and between different levels (Table 4, NUR 15; NUR 3). Educational activities included guidelines for lifestyle change, weight control, smoking cessation, and chronic pain control, most of which were conducted by physicians and nurses.

The use of innovative technologies aimed at screening, diagnosis and blood pressure, and blood glucose control monitoring in people with chronic diseases was also reported in PHC, including a device that measures fingertip glycated hemoglobin and home blood pressure monitoring (HBPM; Table 4, NUR 3, NUR 15).

Healthcare protocols often used to subsidize the therapeutic conduct in several treatment modalities, especially in individual care, were pointed out by medical professionals (Table 4, PHY 10). It was identified that nursing professionals knew the national protocols, which are more commonly used in home visits for chronic patients, since this practice is most performed by these professionals.

Information exchange and case discussion from a multidisciplinary perspective was referred to as a process occurring in meetings and in individual contact with each member of the FHT. Nursing technicians and CHWs were mentioned as those who identify and communicate the users’ needs to the team, as they are in close contact with users inside and outside the units (Table 4, NUR 11, PHY 3). The integration of the FHT with FHSC for the execution of activities of a technical-pedagogical or clinical-assistance nature, presents difficulties for the reasons mentioned above (Table 6).

As for the establishment of a bond with the community, this is possible through the architecture of the FHS, which presupposes it as inherent in the practice of any professional, but also because it advocates that all people who arrive at the service can be welcomed with active and qualified listening by reference professionals located in a specific sector of these units. Nevertheless, physicians reported difficulties due to the high demand for individual care and the consequent reduction in consultation time (Table 4, PHY 5). The nurses reported successful experiences regarding the use of this resources such as home visits, when they had more time to talk to, and to evaluate the living conditions and health needs of NCD patients (Table 4, NUR 5).

Health care longitudinality was expressed as the possibility of periodic follow-up or management of only the most serious NCD cases (Tables 4 and 5, PHY 2). Follow-up strategies through visits, consultations and participation in educational groups occurred according to the perception of necessity/severity due to the need for periodic prescription renewal, since free medication distribution depends on it, as well as according to the severity of the clinical condition evaluated by the team (Table 4, PHY 2, NUR 13).

Physicians and nurses reported that the consistency and completeness of filling data into the electronic medical record (EMR) was hampered by poor Internet connection and constant system updates, which often make the system unavailable. Some professionals also report that the system interface is not friendly or practical for inserting data or for visualization (Table 4, PHY 4, NUR 11). As a positive aspect, all the professionals used the EMR and it had the potential to provide indicators that can contribute to improvements in the provision of care (Table 6).

### Biopsychosocial approach to healthcare practices

The recognition of the psychosocial profile of NCD users by physicians and nurses emerged as the main potentiality to map living and health conditions, and, above all, social and contextual factors located outside the health systems. Low socioeconomic status, an aging population, single-parent households, and family disarray are part of the vulnerability profile of the patients (Table 5, NUR 3; NUR 13). Additionally, the professionals reported their commitment to seeking differentiated and adapted alternatives among the team members themselves and with other sectors, their family and the community, to intervene in disparities that increase the chances of progression morbidity to mortality in user’s with NCD (Table 6). This mobilization was permeated by obstacles related to intersectoral articulation which is still fragmented, little explored and does not solve short-term problems.

Health education actions to explain the importance of lifestyle changes, fostering co-responsibility and self-management in people with chronic diseases during the health-disease-care process were identified to stimulate the users’ self-care and autonomy (Table 5, NUR 2, PHY 10). Educational groups, home visits, and even individual consultations included modalities for the expression of this practice (Table 5).

Physicians and nurses recognize social support and support networks in their communities. It was found that doctors and nurses establish partnerships widely with other actors (community and religious leaders) and with social devices (schools, churches, social assistance referral centers, and residents’ and diabetic patients’ associations) to enhance the development of activities that encourage lifestyle changes, support for community actions, and the strengthening of social and family co-existence (Table 6). However, family support was reported to be challenging due to the lack of support and family participation in the process of taking care of a sick family member (Table 5, NUR 20).

## Discussion

A combined analysis of different dimensions (organizational, technical care and psychosocial) of care practices performed by PHC doctors and nurses in Brazil, is relevant since the combination of constituent elements of models aimed at providing care for chronic conditions can achieve better results in practice [7, 13] to meet the needs of people with NCD. For all analyzed dimensions, strengths and weaknesses were found, which can impact the scope of care practices and; therefore, the management of chronic diseases in a comprehensive and resolute way in PHC.

Regarding access, its guaranteed as a right of every Brazilian citizen and a duty of the state is an essential component of the SUS and contributes to universal health coverage [7, 12]. The diversification of strategies is operationalized to legitimize access to services offered in the FHS as sustained care for NCD is essential to avoid interruptions in treatment, and consequently, poorly managed health conditions, potential complications, and comorbidities [3, 13].

In the FHS investigated, access prevailed based on spontaneous demand, in a complementary way, the flow of some demands through group care access. Group assistance denotes the existence of structural arrangements for the organization and manage the health system and local services due the fragmentation of actions, gaps in care, supply and demand disparities, and lack of professionals [24].

As for supply and demand, discourses about insufficient specialized consultations and examinations seems to be aggravated by the high number of people with chronic diseases requiring follow-up by the teams. Duplication of diagnostic and therapeutic procedures, and unnecessary referrals also represent obstacles to the quality of health systems [8]. In contrast, other international reports highlighted practices in line with recognition of more critical user profiles, monitoring of queues and waiting time for appointments, and the use of decision support systems for health professionals [13, 26, 27].

While guaranteeing access to PHC is essential for coordination of the therapeutic itineraries of users in the services, this is not entirely enough to handle the management of NCDs alone, since articulation with other levels of care may be necessary. The articulation between levels was characterized as precarious confirming PHC difficulties in interacting and communicating with secondary and tertiary levels, which shows the fragility of health care coordination. This is worrisome when considering that people with chronic diseases need continued care and use several devices at different healthcare levels, in turn requiring synchronous coordination of the HCN to guarantee timely and quality assistance [28], but are at greater risk of receiving fragmented care because they are treated by different professionals in diverse contexts [29].

The growing complexity of chronic health care calls for team integration and collaboration. When collaborative primary care practices are based on horizontal work organization and shared decision-making, they can help establish effective and resolutive multidisciplinary teams, with better professional interaction and use of material resources, time optimization, lower rate of patient retention in treatment, and increased work quality and satisfaction [16].

Information exchange and case discussion was widespread practice in the FHT, which contributes to expanding autonomy and self-care, and ratifies the importance of teamwork as a pillar of FHS. Differences observed in the performance of the FHSC in relation to the organization and execution of work in the FHS constituted an important barrier. Despite of the FHSC have being created to increase the resolution and comprehensiveness of care provided in the FHS, your performance can improve. The prioritization of collective activities, restrictions on individual consultations and home visits, and the high turnover between teams were problems that stood out in another study [30–32].

The provision of a wide range of health promotion, prevention, and health care actions, financed by the State, and activated by the population based on individual and collective needs, was widely mentioned. However, although not unanimous, a certain technical work division has emerged as a strong local element, designating the individual care of people with chronic diseases to the physician and collective activities to other professionals, especially nurses.

Although this aspect may be an institutionalized reality in other contexts owing to difficulties in accessing physicians, it is interesting to note that the effectiveness and efficiency of health services [16] in Brazil, in which FHS is traditionally a multidisciplinary work, can translate into fractional practices based on the specialization and fragmentation of knowledge and work and restricting comprehensiveness [33].

Opportunities were identified in services that use multiple care technologies towards providing comprehensive care for NCD. Innovative technologies used in local PHC, like the fingertip glycated hemoglobin tester and home blood pressure monitoring, were identified as facilitators for improving access to glycemic and pressure control, both in screening and therapeutic follow-up actions [34, 35]. The reception and bonding are also recognized as a technology of relationships and advocated in Brazil, as a care practice based on the lasting and authentic relationship, mutual understanding, and sense of affiliation [36], stimulating lifestyle changes, and increasing responsibilities and adherence to treatment [22, 31], however more difficult to operationalize due to the great demand for individual care.

Although system computerization can make health information feasible in a safe, consistent, and relevant manner even between different care levels (which was not observed in this study), EMR implementation is challenging, especially in the context of recent implantation. It needs to be better consolidated and explored considering the need for improvements in the interface and connectivity, as well as for the issuance of reports and indicators that facilitate the planning and monitoring of practices.

Actions stimulating autonomy and self-care are strategic pillars in any health program or system aimed at NCD patients due to the longitudinal character of signs, symptoms, risk factors, and the possibility of related complications. The educational groups were a strategy to increase autonomy and self-care, as reported in another study [37], but other modalities were also highlighted, such as home and individual visits. Moreover, contact by telephone or text messages, as well as actions to stimulate autonomous medication management are also innovative practices highlighted in international studies [13, 27], while collective consultations, physical activity practices, and those involving arts were reported in national studies [23, 38, 39].

There is a worrying trend regarding the impact of socioeconomic inequalities in Latin America on health indicators [40]. In Brazil, inequalities are persistent and systematic, and point a context of health vulnerabilities that affect people with NCDs and can lead to unfavorable clinical outcomes [3]. This situation requires integrated responses from FHT to create synergies between different sectors, actors, and social support devices.

In this study, the integrated and successful responses of the teams dealt with the recognition of the socio-epidemiological profile of the registered area for the recognition of vulnerability profiles that interfere in the control of NCDs and in the mobilization of the social support network located in the community.

However, these answers were unable to account for an important part of this network, which is the family, due to the difficulty of including them as an active participant in the care of users with chronic conditions and which requires integrate interventions by the team. Intersectoral actions were also not very evident, which reflects the need for greater equity and integration of the health sector with other sectors to guarantee people a level of health that allows them to lead a socially and economically productive life [40].

### Strengths and limitations

A strength point of the study was the combinate use of qualitative design and a multidimensional approach to care practices in PHC, which allowed the identification of important insights in the management of NCDs: better results from the practices of physicians and nurses related to elements that depended on their governability, such as those related to organization of access, teamwork, stimulation of autonomy and self-management, and interaction with services and actors in the provision of care. Dependence on structural aspects, especially related to the high demand for people per team, the insufficient quota for specialists, the fragmentation of HCN and problems in the EMR, there was greater compromise in the response, suggesting investments of different orders in this regard.

A limitation of the study is that the data were from the perspective of doctors and nurses (only two professional categories), which may give some specificity to the data. However, as they were responsible for the widest range of actions relative NCD, this favored representation and contributed to their choice in line with the research proposal. The inclusion of only the most prevalent NCD (HBP and DM) also limited the recognition of care practices for other chronic diseases, which may have other specificities.

Therefore, future studies may explore the same object considering other subjects (CHW, users, managers), scenarios (other municipalities and states), designs (quantitative, mixed methods), and the effects of these practices on treatments and blood pressure and glycemic control outcomes.

## Conclusion

The study revealed that the health practices of PHC physicians and nurses presented strengths and weaknesses in the management of chronic conditions, even in a city that is considered a reference in the provision of primary care. This situation confirms the need to develop best practices in PHC as a way of ensuring qualified assistance to people with NCD.

The multidimensional evaluation of practices aimed at people with NCD proved to be effective in demonstrating that all dimensions considered need careful analysis by physicians and nurses, including those that presented more satisfactory results in order to be enhanced. The Brazilian experience can serve both to guide internal changes in the teams’ work process, and to support analyses aimed at formulating health policies to guarantee technical, political, logistical, and financial support in other similar primary care contexts.

## Data Availability

Data that are generated or analyzed during this study are not publicly available to preserve the anonymity of participants, but are available from the corresponding author upon reasonable request.

## Abbreviations

CHW: Community Health Workers
DM: Diabetes Mellitus
EMR: Electronic Medical Record
FHS: Family Health Strategy
FHSC: Family Health Support Centers
FHT: Family Health Team
HBP: High Blood Pressure
HBPM: Home Blood Pressure Monitoring
HCN: Health Care Network
NCD: Chronic Noncommunicable Diseases
NUR: Nurses
PHC: Primary Health Care
PHY: Physicians
Unified Health System: (*Sistema Único de Saúde*-SUS)
